# Salvage chemotherapy with irinotecan, 5-fluorouracil and leucovorin for taxane- and cisplatin-refractory, metastatic gastric cancer

**DOI:** 10.1038/sj.bjc.6602575

**Published:** 2005-05-03

**Authors:** S T Kim, W K Kang, J H Kang, K W Park, J Lee, S-H Lee, J O Park, K Kim, W S Kim, C W Jung, Y S Park, Y-H Im, K Park

**Affiliations:** 1 Division of Hematology and Oncology, Department of Medicine, Samsung Medical Center, Sungkyunkwan University School of Medicine, 50 Ilwon-dong, Kangnam-gu, Seoul, 135-710, Seoul, Korea

**Keywords:** gastric carcinoma, irinotecan, 5-FU, salvage chemotherapy

## Abstract

We performed a phase II study of combination chemotherapy with irinotecan, 5-fluorouracil (5-FU) and leucovorin in metastatic gastric cancer patients who were previously treated with taxane and cisplatin, to evaluate the antitumour activity and toxicity of the combination chemotherapy. The metastatic gastric adenocarcinoma patients who were previously treated with taxane and cisplatin combination as first line, and had at least one measurable lesion, 0–2 ECOG performance status and adequate organ functions, were considered eligible. They received irinotecan (150 mg m^−2^, day 1) and leucovorin (100 mg m^−2^, day 1), followed by continuous infusion of 5-FU (1000 mg m^−2^ day^−1^, days 1 and 2) every 2 weeks. Treatment was continued until progression of disease was observed. In all, 64 patients were treated with this combination chemotherapy. The median age of the patients was 55 years (range, 33–74 years), and the median ECOG performance status was 1 (0–1, 61 (95%)). Out of 64 patients, 57 were assessable for response. Among 57 assessable patients, no complete response and 12 partial responses were observed (overall response rate, 21%; 95% confidence interval (CI), 10–32%). Stable disease was observed in 14 patients (25%) and progressive disease in 31 patients (54%). The median time to progression was 2.5 months (95% CI, 1.6–3.4) and the median overall survival since the start of the second-line modified FOLFIRI was 7.6 months (95% CI, 6.5–8.7). Grade 3–4 haematologic toxicities included neutropenia in seven patients (11%) and thrombocytopenia in five patients (8%). Grade 3–4 nonhaematologic toxicities included diarrhoea in two patients (3%) and vomiting in two patients (3%). There were no treatment-related deaths. The combination of irinotecan, 5-FU and leucovorin showed moderate activity and favourable toxicity profile as a second-line treatment in metastatic gastric cancer patients, who were previously treated with taxane and cisplatin.

Gastric adenocarcinoma is the most common malignancy and one of the major causes of cancer death in Korea (Bae *et al*, 2002). The prognosis of unresectable gastric cancer has been improved by palliative chemotherapy (Murad *et al*, 1993; Pyrhonen *et al*, 1995; Glimelius *et al*, 1997), but median overall survival (OS) rarely exceeds 1 year (Wils, 1998). Studies showed the benefit of combination regimens, such as fluorouracil (FU), doxorubicin and methotrexate (FAMTX) (Wils, 1998) or etoposide, leucovorin (LV) and FU (ELF), over best supportive care (Murad *et al*, 1993; Pyrhonen *et al*, 1995; Glimelius *et al*, 1997). Other combination regimens investigated include epirubicin, cisplatin and infusional FU (ECF) and 5-day infusional FU plus cisplatin (FUP). However, a randomised phase *β* trial showed that there were no significant differences in response rates (RRs) or survival durations among the combination regimens. Although there are no standard first-line chemotherapeutic drugs in unresectable gastric cancer, ECF is generally considered as a standard therapy in Europe after the results from phase III trial have been published (Webb *et al*, 1997).

Taxane and cisplatin has been used as a first-line chemotherapy with various schedules and doses since the late 1990s (Roth *et al*, 2000; Ridwelski *et al*, 2001; Kornek *et al*, 2002; Lee *et al*, 2004). Taxane and cisplatin chemotherapy has produced 30–50% of RRs as a first-line treatment, but the diseases of responding and nonresponding patients eventually progress (Roth *et al*, 2000; Ridwelski *et al*, 2001; Kornek *et al*, 2002; Lee *et al*, 2004). A randomised phase III trial showed a higher RR (39 *vs* 23%) and longer time to progression (TTP; 5.2 *vs* 3.7 months) in DCF (docetaxel, cisplatin, 5-fluorouracil) arm when compared to CF (cisplatin, 5-fluorouracil) arm (Ajani *et al*, 2003).

In addition to novel combinations of established agents, the use of newer agents, such as irinotecan, is under investigation. Irinotecan, a topoisomerase I inhibitor, proved to have a clinical activity against unresectable gastric cancer. The RR of irinotecan alone has been reported to be 20–25% in phase II studies (Futatsuki *et al*, 1994; Kohne *et al*, 2003), regardless of prior chemotherapy. Combination of irinotecan with cisplatin showed the RR of 48% in metastatic gastric cancer patients who had received no or one chemotherapy regimen (Boku *et al*, 1999), and combination with 5-fluorouracil (5-FU) produced the RRs of 22–42.4% in previously untreated gastric adenocarcinoma patients (Blanke *et al*, 2001; Assersohn *et al*, 2004; Bouche *et al*, 2004).

We performed a phase II study of a combination chemotherapy with irinotecan, leucovorin and 5-FU in metastatic gastric cancer patients, who were previously treated with taxane and cisplatin combination, to evaluate the antitumour activity and toxicity of the combination chemotherapy.

## MATERIALS AND METHODS

### Eligibility

Patients were eligible with histologically confirmed, unresectable adenocarcinoma of the stomach. They were required to be between 18 and 75 years, have at least one measurable lesion, Eastern Cooperative Oncology Group (ECOG) performance status ⩽2, and have a life expectancy at least 3 months. Eligible patients must have received one prior palliative chemotherapy with taxane and cisplatin. Adequate haematologic function (absolute neutrophil count (ANC) ⩾1500 mm^−3^, platelet count ⩾100 000 mm^−3^), hepatic function (aspartate aminotransferase/alanine aminotransferase (AST/ALT) ⩽3.0 times the upper normal limit (UNL), bilirubin ⩽1.25 times the UNL), and renal function (serum creatinine ⩽1.5 times the UNL) were required. Prior history of another malignancy within 5 years of study entry, apart from basal cell carcinoma of the skin or carcinoma *in situ* of the uterine cervix, precluded participation in the current trial. Patients with concurrent uncontrolled medical illness were also excluded. All patients provided a written informed consent according to the guideline provided by the institutional review board.

### Chemotherapy

Chemotherapy was administered through a central venous catheter (Hickman catheter or chemoport) placed in the subclavian vein, and ambulatory pumps were used for continuous infusion of 5-FU. Irinotecan (150 mg m^−2^, over 90 min, day 1), followed by leucovorin (100 mg m^−2^, over 2 h, day 1), and then followed by 5-FU (1000 mg m^−2^, over 24 h, day 1 and 2) were given as an intravenous infusion. Cycles were repeated every 2 weeks. Before irinotecan infusion, 0.3 mg atropine was subcutaneously administered to prevent cholinergic syndrome. A 5-hydroxytryptamine type 3 receptor antagonist was given as antiemetic prophylaxis immediately before chemotherapy and loperamide was provided for diarrhoea. Treatment was continued until documented disease progression, unacceptable toxicity, patients' refusal, or physicians' decision.

### Dose modification

Chemotherapy was withheld if the ANC was <1500 mm^−3^ or the platelet count was <100 000 mm^−3^ on day 1. In this case, the complete blood cell count (CBC) was repeated at least weekly and chemotherapy was restarted as soon as the ANC reached ⩾1500 mm^−3^ and platelet count ⩾100 000 mm^−3^. If nadir ANC was less than 500 mm^−3^, or the nadir platelet count was less than 50 000 mm^−3^, the doses of irinotecan and 5-FU were reduced to 120 and 800 mg m^−2^, respectively. If grade 3–4 nonhaematologic toxicity other than alopecia, the treatment was withheld until recovery to grade 0 or 1 and the doses of irinotecan and 5-FU were reduced to 120 and 800 mg m^−2^, respectively. If patients required a delay of longer than 2 weeks, they were treated off the protocol.

### Assessment of efficacy and toxicity

At study entry, the following investigations were performed: full history taking and physical examination, CBC, chemistry, chest X-ray and computed tomography scan. History taking, physical examination, CBC and chemistry were repeated before each cycle. Computed tomography scans were performed to document the disease extent and to evaluate response to treatment, every three cycles and when needed for the confirmation of response and suspected disease progression. Responses were classified according to World Health Organization (WHO) criteria. Patients were considered assessable for response if they had early disease progression or had received at least three cycles of treatment with at least one tumour assessment. Response rate was calculated as the ratio of number of patients who achieved complete responses or partial responses to the number of assessable patients. Duration of response was calculated from the first day of treatment to the date on which progressive disease was first observed or of the last follow-up, for the group of responding patients. Time to progression was calculated from the first day of treatment to the date on which progressive disease was first observed or of the last follow-up. Overall survival was calculated from the first day of treatment to the date of death or last follow-up.

Toxicity was graded according to National Cancer Institute common toxicity criteria (NCI-CTC) version 2.0. (NCI-CTC Web site). The severity of any toxicities not defined in the NCI-CTC were graded as 1=mild, 2=moderate, 3=severe or 4=very severe.

### Statistical analysis

Descriptive statistics were reported as proportions and medians. Kaplan–Meier estimates were used in the analysis of all time-to-event variables, and the 95% confidence interval (CI) for the median time to event was computed. The dose intensity (DI) was calculated as the ratio of the total dose (expressed in milligrams) per square meter of the patient, divided by the total treatment duration expressed in days. In this calculation, the end of treatment was considered to be 14 days after day 1 of the last cycle of chemotherapy. The relative DI was calculated as the ratio of the DI actually delivered to the DI planned by the protocol.

According to Simon's two-stage optimal design, a sample size of 55 was required to accept the hypothesis that the true RR is greater than 25% with 90% power, and to reject the hypothesis that the RR is less than 10% with 5% significance. At the first stage, if there were fewer than four responses out of the initial 31 patients, the study would terminate. Although the target number of patients was 55, we planned to recruit 20% more than the target number of patients considering dropout.

SPSS for Windows (SPSS Inc., Chicago, IL, USA) was used for statistical analysis.

## RESULTS

### Patient characteristics

From November 2001 to July 2003, 64 patients were enrolled. The clinical characteristics of enrolled patients are shown in Table 1. The median age of the patients was 55 years (range, 33–74 years) and the majority (73%) of patients was male. The median ECOG performance status was one (0–1, 61 (95%)), and all patients had metastatic disease at study entry. Major involved organs were liver and intra-abdominal lymph nodes. Regimens of the prior chemotherapy are listed in Table 1, and all of theses patients showed progressive disease after taxane and cisplatin before entry into this study.

### Delivery of drugs

The patients received a median of six (range, 1–9) cycles. The average relative dose-intensities were 0.87 for irinotecan, 0.87 for 5-FU and 0.89 for leucovorin. Dose reduction was required in 11 patients (37 cycles) and treatment was delayed in 23 patients (59 cycles). The most common causes of dose reduction were neutropenia (64%), thrombocytopenia (45%), diarrhoea (18%) and vomiting (18%).

### Response rate

In all, 57 patients were assessable for treatment response. Seven patients were excluded from the response analysis; four patients received one cycle and three patients two cycles of chemotherapy. Five of seven patients excluded from the analysis were lost to follow-up and the remaining two patients refused further treatment due to asthenia. Among 57 assessable patients, no complete response and 12 partial responses were observed (overall RR, 21%; 95% CI, 10–32%). Stable disease was observed in 14 patients (25%) and progressive disease in 31 patients (54%) (Table 2). The median duration of response was 5.8 months (95% CI, 4.0–7.6). The differences in RRs were not observed according to baseline patients' characteristics (age, sex, performance status, involved organs and previous chemotherapy regimen).

### Survival

**Figure 1:**
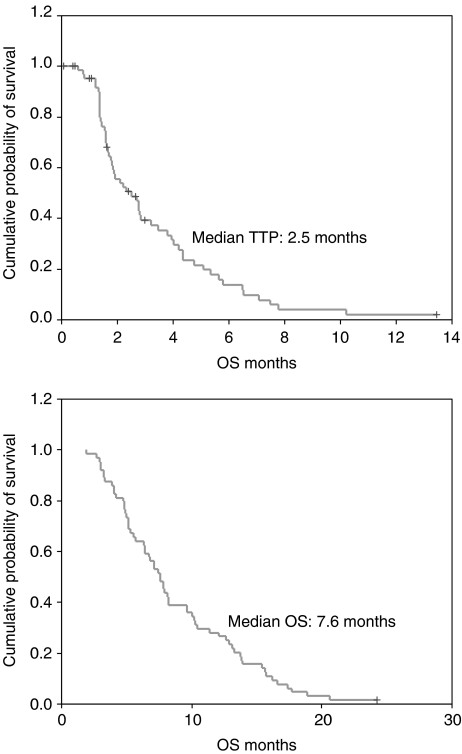
Time to progression and OS.

All 64 patients were in included in the survival analysis on an intent-to-treat basis. The median follow-up time was 22 months (95% CI, 20–25). The median TTP was 2.5 months (95% CI, 1.6–3.4) and the median OS was 7.6 months (95% CI, 6.5–8.7). The differences in TTP and OS were not observed according to age, sex, performance status, involved organs and previous chemotherapy regimen. The TTP and OS curves are shown in Figure 1.

### Toxicity

Toxicities observed during the treatment are listed in Table 3. NCI-CTC grade 3 or 4 haematologic toxicities included neutropenia in seven patients (11%) and thrombocytopenia in five patients (8%). Two patients (3%) with neutropenic fever and four patients (6%) with grade 3 or 4 diarrhoea/vomiting required hospital admissions. There were no treatment-related deaths.

## DISCUSSION

The prognosis of unresectable gastric cancer has remained poor despite new palliative treatment modalities. Response rates as a frontline of various combination chemotherapy regimens ranged between 30 and 70%, but median OS rarely exceeded 10 months (Murad *et al*, 1993; Pyrhonen *et al*, 1995; Glimelius *et al*, 1997; Wils, 1998). Taxane and cisplatin combination chemotherapy regimen has been increasingly used as a first-line treatment in unresectable gastric cancer. Phase *α* studies with paclitaxel or docetaxel in combination with cisplatin with or without 5-FU have demonstrated RRs of 37–51% and OSs of 9–14 months in chemotherapy-naïve patients (Kollmannsberger *et al*, 2000; Ridwelski *et al*, 2001; Polee *et al*, 2002). Other phase II studies have employed the combination of irinotecan and cisplatin; RRs have ranged from 28 to 59% in chemotherapy-naïve patients, with corresponding OSs of 9–11 months (Shirao *et al*, 1997; Boku *et al*, 1999; Pozzo *et al*, 2001; Ajani *et al*, 2002). As most of the patients with unresectable gastric cancer eventually progress after the first-line chemotherapy, the second-line chemotherapy should be developed without cross-resistance to first-line chemotherapy. We studied a new second-line combination regimen, the modified FOLFIRI (irinotecan, leucovorin and 5-FU), in patients who had failed to taxane and cisplatin combination. We had used the reduced dose of irinotecan and 5FU as would be routinely used in similar regimens as in advanced colorectal cancer (i.e. the FOLFIRI regimen). The low dose of irinotecan (150 mg m^−2^, every 2 weeks) used in our study was based on the previous phase *α* studies conducted in Japan (Futatsuki *et al*, 1994; Bleiberg, 1999), which demonstrated modest antitumour activities in unresectable gastric cancer patients with irinotecan 150 mg m^−2^ every 2-week schedule. This regimen showed a RR of 21% in assessable patients and of 19% in all patients as a second-line treatment. In this phase II study, the median TTP was 2.5 months (95% CI, 1.6–3.4 months) and the median OS was 7.6 months (95% CI, 6.5–8.7 months). A previous study of irinotecan, 5-FU and leucovorin against gastric cancer yielded a RR of 22–40% and OS of 7.6–11.3 months as a first-line treatment (Blanke *et al*, 2001; Bouche *et al*, 2004). This three-drug combination regimens with different treatment scheme showed a RR of 29% and an OS of 6.4 months as a second-line treatment against oesophageal and gastric cancer (Assersohn *et al*, 2004).

The regimen showed a favourable toxicity profile. There were no treatment-related deaths. Although haematologic toxicity was reported, the incidence of grade 3–4 haematologic toxicity was approximately 10% (neutropenia in 11% and thrombocytopenia in 8%). NCI-CTC grades 3 or 4 nonhaematologic toxicities were minimal in incidence and all manageable (diarrhoea in 3% and vomiting in 3%). Two previous studies using a similar regimen in gastric cancer showed 36% (Blanke *et al*, 2001) and 26% (Assersohn *et al*, 2004) of grade 3–4 neutropenia, and 28% (Blanke *et al*, 2001) and 8% (Assersohn *et al*, 2004) of grade 3–4 diarrhoea. The significantly low rate of grade 3–4 toxicities in this study when compared to other irinotecan-based trials may be explained by the ethnic difference or the continuous infusion of 5-FU or the dose intensity of irinotecan. The ethnic difference in tolerability may be explained by the UDP-glucuronosyltranferase 1A1 genotype, predictive factor of severe neutropenia (Innocenti *et al*, 2004). In addition, Blanke *et al* used 5-FU bolus, whereas we used continuous infusion of 5-FU. The dose intensities of irinotecan in the previous studies were 83 mg m^−2^ per week (125 mg m^−2^ per week in the first 4 weeks) (Blanke *et al*, 2001) and 90 mg m^−2^ per week (Assersohn *et al*, 2004), but the dose intensity in this study was 75 mg m^−2^ per week.

Based on a comparable activity and a favourable safety profile to other second-line regimens, the combination chemotherapy with irinotecan, 5-FU and leucovorin should be considered as a second-line treatment in taxane- and platinum-treated, unresectable gastric cancer.

## Figures and Tables

**Table 1:** Patient characteristics

**Patient characteristics**	**No. of patients (*N*=64)**	**%**
*Age, years*		—
Median (range)	55 (33–74)	
		
*Sex*		
Female	17	27
Male	47	73
		
*ECOG status*		
0	3	5
1	58	90
2	3	5
		
*First-line regimen*		
Paclitaxel/CDDP	8	14
Docetaxel/CDDP	33	50
EDP	23	36
		
*Organ involved*		
Lymph nodes	34	41
Liver	29	35
Peritoneum	6	7
Ovary	5	6
Others	4	5

Others: pancreas (two), bone (two), lung (one), adrenal (one), uterus (one), ureter (one).

CDDP, cisplatin; EDP, epirubicin/docetaxel/cisplatin.

**Table 2:** Response rate

**Response**	**Number of patients (%)**
Complete response	0
Partial response	12 (21%)
Stable disease	14 (25%)
Progressive disease	31 (54%)
Total assessable patients	57

**Table 3:** Grade 3/4 adverse events (*N*=64)

**Toxicity**	**Number of patients (%)**
Neutropenia	7 (11%)
Thrombocytopenia	5 (8%)
Diarrhoea	2 (3%)
Vomiting	2 (3%)
Total enrolled patients	64
